# How do the 2022 European Society of Cardiology/European Respiratory Society guidelines modify the diagnosis of portopulmonary hypertension in patients with cirrhosis complicated by portal hypertension? A post hoc analysis

**DOI:** 10.1002/jgh3.12949

**Published:** 2023-07-20

**Authors:** Masanori Atsukawa, Akihito Tsubota, Yuichi Tamura, Kaori Koyano‐Shioda, Tadamichi Kawano, Tomomi Okubo, Korenobu Hayama, Taeang Arai, Norio Itokawa, Yu Taniguchi, Yudai Tamura, Chisa Kondo, Katsuhiko Iwakiri

**Affiliations:** ^1^ Division of Gastroenterology and Hepatology Nippon Medical School Tokyo Japan; ^2^ Core Research Facilities The Jikei University School of Medicine Tokyo Japan; ^3^ Pulmonary Hypertension Center International University of Health and Welfare Mita Hospital Tokyo Japan; ^4^ Division of Cardiovascular Medicine, Department of Internal Medicine Kobe University Graduate School of Medicine Kobe Japan

**Keywords:** liver cirrhosis, portal hypertension, portopulmonary hypertension, pulmonary arterial pressure, pulmonary vascular resistance

## Abstract

The vertical and horizontal broken lines indicate the pre‐revised criteria, whereas the vertical and horizontal solid lines indicate the 2022 European Society of Cardiology/European Respiratory Society criteria.

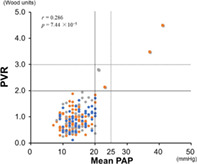

## Introduction

Portopulmonary hypertension (PoPH) is a subtype of pulmonary arterial hypertension (PAH), classified as group 1 pulmonary hypertension (PH).^1^ Recently, the 2022 European Society of Cardiology (ESC)/European Respiratory Society (ERS) guidelines revised the hemodynamic definition of pre‐capillary PH by lowering the mean pulmonary arterial pressure (mPAP) threshold from ≥25 to >20 mmHg and the pulmonary vascular resistance (PVR) threshold from >3 to >2 Wood units (WU).^1^ The mPAP threshold was revised because the normal mPAP is 14.0 ± 3.3 mmHg with an upper limit of 20 mmHg, and patients with mPAP of 21–24 mmHg do not fulfill the PH diagnosis criteria but fall outside the normal limits.^2^ Moreover, in patients with liver cirrhosis, especially those complicated by portal hypertension, cardiac output (CO) and pulmonary arterial wedge pressure (PAWP) increase; thus, a lower PVR could be estimated on the following formula: PVR = (mPAP − PAWP)/CO. Such patients generally have hyperdynamic circulation and impaired pulmonary circulation due to several causes, such as decreased peripheral vascular resistance and portosystemic and intrapulmonary shunt.^3^ Additionally, one study reported that 13 of 16 untreated patients with liver cirrhosis with mPAP >20 mmHg and PVR between 2 and 3 WU progressed to PVR >3 WU during the 5‐year follow‐up period, suggesting that the 2022 ESC/ERS guidelines are useful for the earlier detection of PoPH or potentially progressing conditions in patients with cirrhosis.^4^

Accordingly, the 2022 ESC/ERS guidelines will increase the number of PoPH cases. However, real‐world data on the extent to which patients with PoPH are diagnosed after the publication of the 2022 ESC/ERS guidelines are still limited. Previously, we retrospectively studied 186 patients with liver cirrhosis who underwent hepatic venous catheterization and right heart catheterization simultaneously for diagnosing portal hypertension and PoPH. Among these patients, two (1.1%) were diagnosed with PoPH according to the pre‐revised criteria.^3^

In this post hoc study, we aimed to investigate how the 2022 ESC/ERS guidelines modified the diagnosis of PoPH in these patients with liver cirrhosis complicated by portal hypertension.

## Methods

### Patients

Among 338 patients with liver cirrhosis who underwent hepatic vein catheterization for a definitive diagnosis of portal hypertension at the Nippon Medical School, 186 patients were diagnosed with portal hypertension and simultaneously underwent right heart catheterization, as previously reported.^3^ The inclusion criteria were (i) liver cirrhosis diagnosed through imaging (abdominal computed tomography and/or ultrasonography) or liver biopsy; (ii) hepatic vein pressure gradient ≥5 mmHg; and (iii) patient consent to simultaneously undergo right heart catheterization using the Swan‐Ganz catheter for diagnosing PoPH. The exclusion criteria were (i) uncontrollable hepatocellular carcinoma, that is, hepatocellular carcinoma that has not been cured by operation, radiofrequency ablation therapy, and transcatheter arterial chemoembolization, and so on; (ii) chronic respiratory disease; (iii) left ventricular heart disease; (iv) chronic pulmonary embolism; (v) blood disease; (vi) sarcoidosis; (vii) thyroid disease; (viii) connective tissue disease; (ix) human immunodeficiency virus infection; and (x) congenital heart disease. Hepatic vein and right heart catheterizations were performed as previously reported.^3^

### Conventional and newly proposed diagnostic criteria of PoPH

Conventionally, PoPH was defined as the presence of portal hypertension, mPAP ≥ 25 mmHg, PVR > 3 WU, and PAWP ≤ 15 mmHg according to the pre‐revised diagnostic criteria. Meanwhile, according to the newly proposed definition, PoPH is the presence of portal hypertension, mPAP > 20 mmHg, PVR > 2 WU, and PAWP ≤ 15 mmHg based on the 2022 ESC/ERS guidelines.^1^

### Ethical statement

This study was conducted in accordance with the ethical guidelines established in the 2013 Helsinki Declaration, and was approved by the ethics committee of the Nippon Medical School (approval No. B‐2019‐098). Patients were given the option to abstain from participating in this retrospective study.

### Statistical analyses

Continuous variables are presented as medians and ranges. Categorical variables are presented as numbers. Spearman's rank correlation coefficient was used to evaluate the association between mPAP and PVR. All statistical analyses were performed using SPSS version 17.0 software (IBM Japan, Tokyo, Japan). The level of significance was set at a *P*‐value of <0.05.

## Results

### Characteristics of patients

The patient population comprised 138 males and 48 females, with a median age of 59 (range 35–80) years, as previously described.^3^ The etiologies of liver cirrhosis were alcoholic liver disease (*n* = 61), chronic hepatitis B (*n* = 11), chronic hepatitis C (*n* = 91), autoimmune hepatitis (*n* = 2), primary biliary cholangitis (*n* = 4), non‐alcoholic steatohepatitis (*n* = 7), and others (*n* = 10). Child–Pugh classes A, B, and C accounted for 53, 92, and 41 patients, respectively. The median mPAP, PVR, PAWP, and CO were 13 (range 7–41) mmHg, 0.8 (range 0.1–4.5) WU, 8 (range 3–15) mmHg, and 6.7 (range 2.8–14.7) L/min, respectively.

### Distribution of pulmonary hemodynamics parameters according to the conventional and newly proposed diagnostic criteria

**Figure 1:**
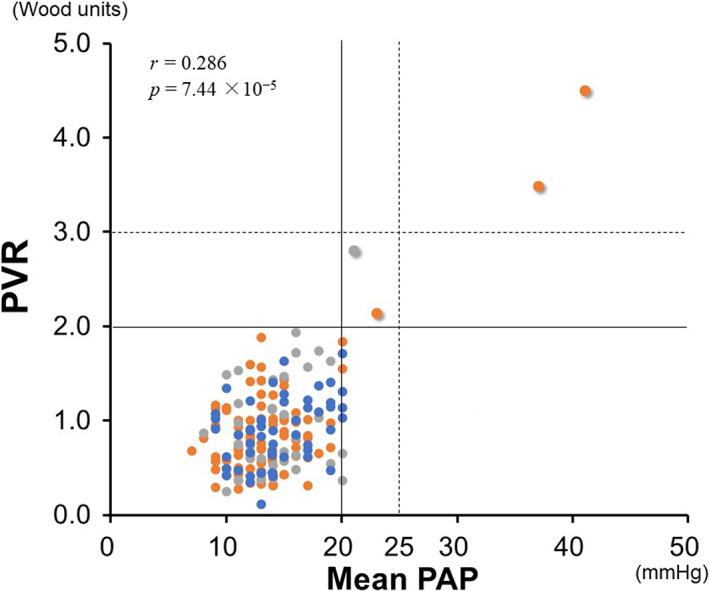
Correlation between mean pulmonary artery pressure and pulmonary artery resistance values in 186 patients with liver cirrhosis complicated by portal hypertension. Blue, orange, and gray circles denote patients with Child–Pugh class A, B, and C, respectively. The vertical and horizontal broken lines indicate the pre‐revised criteria, whereas the vertical and horizontal solid lines indicate the 2022 ESC/ERS criteria. (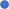), Child–Pugh class A; (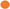), Child–Pugh class B; (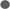), Child–Pugh class C. PAP, pulmonary arterial pressure; PVR, pulmonary vascular resistance.

Two (1.1%) out of the 186 patients had conventional PoPH, as reported previously.^3^ According to the 2022 ESC/ERS guidelines, two patients (without conventional PoPH) were newly diagnosed with PoPH. One was a 65‐year‐old female with autoimmune hepatitis (Child–Pugh class B, 8 points), and the other was a 70‐year‐old male with alcoholic cirrhosis (Child–Pugh class C, 10 points). mPAP, PVR, and PAWP were 23 mmHg, 2.1 WU, and 12 mmHg for the former, and 21 mmHg, 2.8 WU, and 10 mmHg for the latter, respectively. Thus, four (2.2%) patients had PoPH. The remaining 182 had both mPAP ≤20 mmHg and PVR ≤2 WU, and they were not diagnosed with PoPH. No patients met the criterion for either mPAP or PVR, or both diagnostic criteria. All patients were categorized into two groups: those who met both criteria (upper right in Fig. 1) or those who met neither criterion (lower left in Fig. 1).

## Discussion

This post hoc study assessed the clinical implications of the 2022 ESC/ERS guidelines of PAH for patients with liver cirrhosis complicated by portal hypertension. These guidelines increased PoPH prevalence from 1.1 to 2.2% in this patient cohort, suggesting that such guidelines may facilitate earlier identification of PoPH. Of note, no patients met the criterion of mPAP >20 mmHg alone or PVR >2 WU alone. As shown in Figure 1, mPAP significantly but weakly correlated with PVR (*P* = 7.44 × 10^−5^, *r* = 0.286). Echocardiography is recommended as the first noninvasive assessment approach for patients with portal hypertension or portosystemic shunt with suspected PH.^1^ Given that tricuspid regurgitant pressure gradient (TRPG) on echocardiography correlates with mPAP, patients with high TRPG should undergo right heart catheterization. As shown in this study, all patients with PoPH with mPAP >20 mmHg had PVR >2 WU, thereby fulfilling the 2022 ESC/ERS criteria for PAH. Thus, TRPG measurement on echocardiography may be a critical surrogate indicator for screening for PoPH. According to a previous study,^5^ the cut‐off mPAP value of 25 mmHg on right heart catheterization corresponds approximately to the cut‐off TRPG value of 35 mmHg on echocardiography. We have reported that TRPG ≥35 mmHg is associated with female sex, shortness of breath, and brain natriuretic peptide (BNP) ≥48.9 pg/mL and suggested that patients with these factors should undergo echocardiography.^6^ However, this cut‐off TRPG value needs to be re‐established with the publication of the 2022 ESC/ERS criteria.

The prevalence of PoPH in patients with liver cirrhosis or portal hypertension is 1–6%.^3^, ^7^, ^8^, ^9^ In this study, the prevalence was 1.1% using the conventional diagnostic criteria and 2.2% using the 2022 ESC/ERS guidelines, consistent with the previously reported prevalence rates. Intriguingly, none of the patients met either the mPAP or PVR component alone or both diagnostic criteria, suggesting that the newly revised mPAP and PVR thresholds enhance earlier detection of latent PoPH. However, this study lacks data on the effect of treatment on PoPH and patient prognosis. Given that cirrhosis with portal hypertension is highly prevalent worldwide (including Japan), the total number of patients with PoPH is too large to ignore.

A strong point of this post hoc study is that it used data that included patients who had undergone hepatic venous pressure catheterization and right heart catheterization simultaneously. To our knowledge, there is no such (relatively) large cohort in Japan. However, this post hoc study lacks data on the effect of treatment for PoPH and prognosis of patients with and without conventional and newly diagnosed PoPH. In fact, the two patients diagnosed with PoPH by the new criteria died early because of refractory ascites. In addition, several background factors such as TRPG and BNP of the newly diagnosed patients were difficult to obtain because they were quite early patients. Future studies are required to address and clarify these issues along with the publication of the 2022 ESC/ERS criteria.

## Conclusions

The 2022 ESC/ERS guidelines identified patients with newly proposed PoPH in addition to those with conventional PoPH, thereby increasing the number of patients diagnosed with PoPH from 1.1 to 2.2% in this cohort. Further research is needed to elucidate how the 2022 ESC/ERS guidelines modify the clinical features and prognosis of patients with PoPH.

## Data Availability

The data underlying this article will be shared on reasonable request to the corresponding author.
